# Fusion Protein Technology to Enhance Pharmacological Properties of L-Asparaginases

**DOI:** 10.3390/biom16070963

**Published:** 2026-06-30

**Authors:** Anastasiya N. Shishparenok, Varvara G. Blinova, Dmitry D. Zhdanov

**Affiliations:** Laboratory of Medical Biotechnology, Institute of Biomedical Chemistry, 10/7 Pogodinskaya St., 119121 Moscow, Russia; a.shishparyonok@yandex.ru (A.N.S.); varya.blinova@list.ru (V.G.B.)

**Keywords:** fusion protein, L-asparaginase, half-life, cytotoxicity

## Abstract

L-asparaginase (L-ASNase) is a key therapeutic enzyme used in the treatment of acute lymphoblastic leukemia and other hematological malignancies. However, its clinical application is limited by a short plasma half-life, significant toxicity, and immunogenicity. To address these limitations, various strategies have been developed, including conjugation of the enzyme with polyethylene glycol and the use of enzymes from alternative sources with lower immunogenicity. Nevertheless, effective targeting of tumor cells, particularly in solid tumors, remains a major challenge. Protein fusion technology has emerged as a promising approach to improve the pharmacological properties of L-asparaginase by enhancing stability, prolonging circulation time, enabling targeted delivery, and integrating multiple functional domains into a single construct, thereby addressing several limitations simultaneously. This review analyzes current strategies for the design of L-asparaginase-based fusion proteins, including the fusion of protein domains to improve pharmacokinetics and the fusion of targeting peptides or proteins to enhance local cytotoxicity. A comparative analysis indicates that elastin-like peptide (ELP)-based constructs primarily enhance the half-life of L-ASNase, whereas albumin-binding domain (ABD)- and heparin-binding domain (HBD)-based fusions provide more pronounced improvements in both half-life extension and in vivo efficacy. However, described strategies require further validation to ensure enhanced selectivity. Overall, fusion protein technology represents a promising avenue for the development of next-generation L-asparaginase therapeutics.

## 1. Introduction

The rapid evolution of biopharmaceutical technologies has driven research into novel and enhanced therapeutics, including L-ASNase [1]. L-ASNase is a chemotherapeutic enzyme included in the World Health Organization’s List of Essential Medicines. It is used to treat acute lymphoblastic leukemia (ALL) [2,3], the most common pediatric malignancy and a leading cause of cancer-related morbidity and mortality in children and adolescents [4]. The mechanism of action of L-ASNase involves the breakdown of extracellular L-asparagine. Unlike normal cells, ALL cells and some other tumor cells cannot synthesize enough L-asparagine due to low asparagine synthetase expression. Consequently, a deficiency of this amino acid leads to their death [5].

L-ASNases approved for clinical use include those from Escherichia coli (*E. coli*, EcA II; Spectrila^®^, Onconase^®^) and *Erwinia chrysanthemi* (ErA II; Erwinaze^®^, Rylaze^®^) and their polyethylene glycol (PEG) conjugate forms (Asparlas^®^, Oncospar^®^, and Pegaspargase^®^ for EcA II, and Crisantaspase^®^ for ErA II) (Table 1) [6,7,8].

Despite the effectiveness of L-ASNases in treating ALL, their short half-life in the bloodstream and associated side effects—including thrombosis, atherosclerosis, pancreatitis, acute hyperglycemia, hemorrhage, hypersensitivity and the formation of anti-enzyme antibodies—limit their clinical use [10]. To address these drawbacks, various protein modification strategies have been developed [19].

The most common modification of L-ASNase to improve its clinical performance is immobilization on various carriers, particularly polyethylene glycol (PEG). Among these strategies, PEGylation is the most widely used approach and significantly prolongs the half-life of L-ASNases. However, PEGylated L-ASNases have several disadvantages, including a lack of tumor selectivity, the development of resistance, immunogenicity [7], and the absence of metabolic degradation pathways for PEG [20]. Other strategies to enhance L-ASNase efficacy include combination therapy with signaling pathway inhibitors, such as MEK inhibitors [21] or GCN2 inhibitors [22]. However, data on this approach are currently limited to cell lines and a few in vivo studies [21]. One of the most promising strategies for improving the properties of L-ASNases is fusion protein technology. This technology enables functional domains to be combined to enhance stability, targeting, or catalytic activity. Compared with other approaches for generating proteins with novel properties, protein fusion technology offers several distinct advantages: (1) it can facilitate targeted delivery, including approaches aimed at overcoming biological barriers such as the blood–brain barrier [19]; (2) it simplifies the isolation of target proteins [23]; (3) it facilitates the assembly of multi-enzyme complexes [24]; (4) it reduces the number of steps required for protein production and purification; (5) it improves the yield and solubility of recombinant proteins; (6) it allows multiple functional domains to be combined within a single construct [25]; and (7) it can enhance catalytic efficiency [24].

To date, fusion proteins have been approved for treating immunological, oncological, and ophthalmological conditions, as well as diabetes and blood clotting disorders [26,27]. Notable examples include Enbrel^®^ (autoimmune diseases), Ontak^®^ (leukemia and lymphoma), and Amevive^®^ (chronic psoriasis) [28]. An anti-VEGF chimeric antibody has also been developed to treat vascularized solid tumors [29]. Several fusion proteins are currently being evaluated in clinical trials, such as JS014, which was tested in combination with pembrolizumab for cancer therapy (NCT05296772) [30]. Additionally, fusion enzymes have been developed for various applications, including cytochrome P450 fused to its redox partners [7] and Baeyer–Villiger monooxygenases fused with alcohol dehydrogenases or transaminases [5].

The development of L-ASNase-based fusion proteins began in the early 1990s [31], and several promising constructs have been developed to date—these are the focus of this review.

## 2. Design of Fusion Proteins

### 2.1. General Strategies for Producing Fusion Proteins

Fusion proteins typically consist of an effector domain and a functional carrier protein. The effector domain can enhance binding selectivity and increase toxicity [32]. In a fusion protein, one partner in a fusion protein usually handles molecular recognition, while the other improves stability, half-life, and other pharmacological properties—such as cytotoxic effects—or enables novel delivery routes and functions [28]. When designing a fusion gene, the DNA sequence encoding the functional domain can precede or follow the sequence encoding the carrier protein, provided there are no stop codons interrupting the junction between them. The order of the proteins within a polypeptide chain can significantly impact functionality [28].

**Figure 1:**
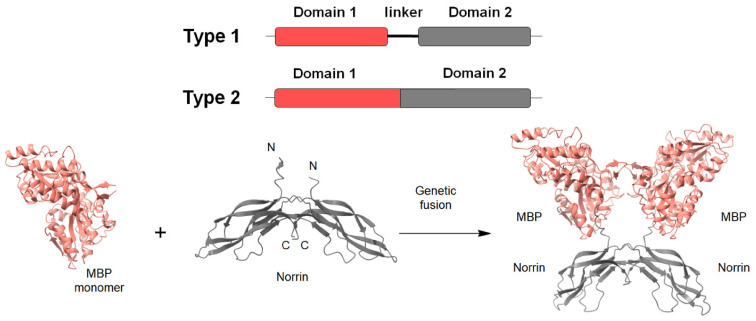
Schematic of the tandem fusion strategy. Two proteins may be joined either with or without a linker peptide. The schematic structure of MBP fused to norrin (PDB: 4MY2) via tandem fusion is shown below. All protein structure figures were prepared using ChimeraX version 1.9.

**Figure 2:**
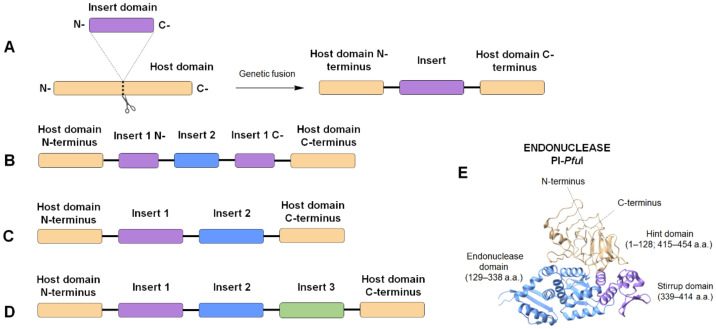
Schematic representation of domain insertion types. (**A**) Single insertion. The domain of one protein is inserted into the domain of a second protein at a recombination site. (**B**) Nested insertion. “Insert 1 N′ terminus” and “Insert 1 C′ terminus” denote the N and C termini of the insert, respectively. (**C**) Two domain insertions. (**D**) Three domain insertions. (**E**) Crystal structure of the endonuclease PI PfuI obtained via two domain insertions (PDB ID: 1DQ3).

**Figure 3:**
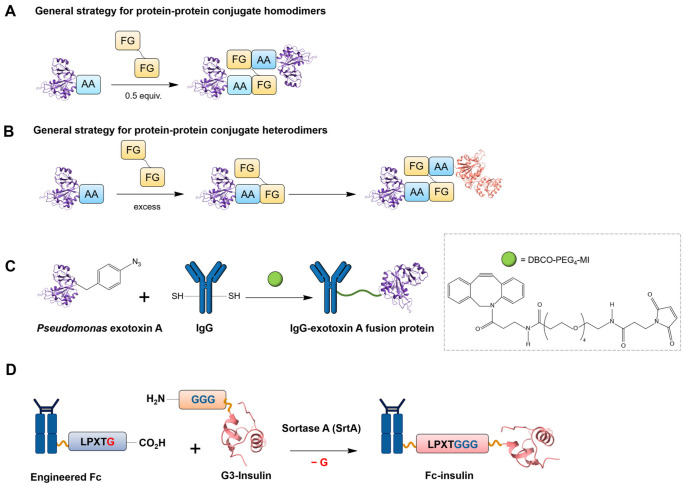
Post-translational conjugation. Separately expressed proteins are joined together to form a new fusion protein using reagents or enzymes. (**A**) Strategy for obtaining fusion proteins using homobifunctional linkers. Homobifunctional linkers target identical amino acid residues in each protein domain. The linker is used in a limited amount. “Equiv.” is the abbreviation for “equivalent”; “FG” stands for “functional group” on the linker responsible for reaction specificity; and “AA” stands for “amino acid residue” on the protein with which the FG reacts. (**B**) Strategy for obtaining fusion proteins using heterobifunctional linkers. The linker is added in molar excess. (**C**) Preparation of an IgG-exotoxin A fusion protein via maleimide (MI) click chemistry using dibenzocyclooctyne (DBCO) attached to an engineered cysteine residue. (**D**) Enzymatic method. Schematic representation of the sortase-based approach. Sortase catalyzes cleavage of the amide bond at the LPXT↓G motif (where X represents any amino acid) and ligates the N-termini polypeptide to a nucleophile (polyglycine residues). The C-termini glycine residue in the LPXTG motif is released, forming a new intermolecular peptide bond [45]. The figure shows a schematic representation of Fc-insulin fusion protein conjugation [46].

There are several common strategies that exist for producing fusion proteins: tandem fusion (Figure 1), domain insertion (Figure 2), and post-translational conjugation (Figure 3). Tandem fusion relies on direct fusion when sufficient space exists between the protein domains to allow proper folding (Figure 1). However, this approach can be ineffective if the free N-terminus or C-terminus is essential for function, or if it lacks the necessary flexibility or length to avoid misfolding and aggregation. These limitations can be overcome by adding a peptide linker between the protein domains [28]. Tandem arrangements are common in bispecific antibody fusions, including approved therapeutics. For instance, blinatumomab (Blincyto^®^), a drug used to treat ALL, links an anti-CD19 single-chain variable fragment (scFv) to an anti-CD3 scFv via a flexible linker [33]. Figure 1 shows the structure of maltose-binding protein (MBP) fused to norrin, which is an example of a protein obtained by tandem fusion for which a known structure exists. Using MBP as a fusion partner increased norrin’s solubility and facilitated structure determination [34].

Domain insertions can be either single or multiple (see Figure 2). In a single insertion, one domain is inserted into another. The domains may belong to the same protein or to different proteins [35]. Sites within a protein that are intrinsically disordered or partially flexible are more suitable for the in-frame of another domain. When producing fusion proteins by domain insertion, the sequences of the protein domains are usually arranged head-to-tail. However, in some cases, the sequence of one protein is inserted into the sequence of a second protein. The resulting fusion protein acts as a switch because this design enables communication between the domains through coupled conformational changes. A major limitation of this approach is the difficulty of identifying suitable insertion sites, since domain fusion can disrupt protein structure and functionality [23]. This limitation stems from the limited structural and functional data available for many proteins [36]. Furthermore, sites where insertion can occur may not be specific to the type of domain being inserted. Consequently, the most promising sites are specific flexible loops on the surface of protein [23].

The domain insertion strategy is a common approach in the engineering of enzymes (e.g., Cas9 [37]), ion channels, and ligand or metabolite sensors [38]. Several insertion domains have been employed to regulate the allosteric activity of proteins, including LOV2, FKBP12, calmodulin, the estradiol-binding domain, nanobodies, and yellow fluorescent protein [39]. In a recent study, a chimeric antigen receptor (CAR) T-cell receptor was developed using a domain-insertion strategy by inserting P7 and N6 peptides into the heavy chain loop of scFv using a domain insertion strategy [40].

The post-translational conjugation method (Figure 3) is used to create fusion proteins that cannot be obtained using the first two strategies. This method is particularly useful when the protein is toxic to a given host organism during expression. Post-translational conjugation relies on the modification of canonical or non-canonical amino acids within proteins. Common strategies include: (1) reduction of disulfide bonds using homobifunctional reagents, such as o-phenylenedimaleimide, bismaleimide, or 2,3-dibromomaleimide (DBM), or click chemistry (Figure 3A); and (2) the use of heterobifunctional reagents, including vinylphosphonates or palladium-protein oxidative addition complexes (Pd-protein OACs) (Figure 3B). For homobifunctional coupling, cysteine–maleimide conjugation is commonly employed, representing one of the most reliable strategies in chemical biology. This approach is often used to produce bispecific antibodies. For example, click chemistry was applied to generate the bispecific antibody HER2 × CD3, which exhibits tumor cytotoxicity [41]. Post-translational conjugation also employs lysine-targeting reagents—such as N-hydroxysuccinimidyl (NHS) esters [42]—as well as metal-mediated conjugation strategies and reagents that target alternative amino acid residues or incorporate unnatural amino acids. These strategies are also used to generate bispecific antibodies [28,43]. Both homo- and heterobifunctional linkers can be used to incorporate unnatural amino acids. These linkers selectively react with natural amino acids on one protein and with engineered residues in another. This approach has been used, for example, to create IgG fusion proteins with immunotoxins [43].

Another approach to post-translational protein conjugation is enzymatic conjugation [28]. These modifications occur at the functional groups of amino acid side chains and include the addition of organic molecules to these groups, intramolecular transformations (e.g., disulfide bond formation and proteolysis), and the formation of covalent bonds between protein molecules [44]. Examples of enzymes used for this process include sortase, tyrosinase, the E2 small ubiquitin-like modifier (SUMO)-conjugating enzyme (Ubc9), lipoic acid protein ligase, and others. Sortase (Figure 3D), for instance, is used to produce N-N and C-C linked protein heterodimers that cannot be generated by genetic engineering alone. Sortase has also been employed to produce bispecific antibodies with broad-spectrum activity against the influenza virus [43].

The main advantage of post-translational conjugation is its ability to arrange proteins in N-to-C-termini, N-to-N, and C-to-C-termini orientations—something that is impossible to achieve with other fusion protein production methods. Another advantage is the ability to perform conjugation at a late stage of the production process. The main disadvantages of this method are: (1) the frequent lack of selectivity when modifying the target sequence of the fusion protein; (2) the potential immunogenicity of the linker; (3) the complexity in assessing product yield; and (4) the limited abundance of available cysteine residues [43].

Recently, bioinformatics tools have been used to design fusion proteins and predict their properties. AlphaFold, RoseTTAFold, trRosetta, and D-I-TASSER are used for structure prediction, while molecular dynamics simulations employ packages such as GROMACS, AMBER, NAMD, and CHARMM [47]. For instance, an antiangiogenic fusion protein was designed using bioinformatics tools by connecting the soluble extracellular domain of human sVEGFR-1 to human IL-2 via a flexible linker using bioinformatics tools [48]. In addition, in silico approaches have been used to construct the chimeric fusion proteins IL24-LK6 [49] and Azurin-BR2 [50] as potential candidates against breast cancer. Bioinformatics methods have also been applied to create immunotoxins for targeted cancer therapy. The structures of these fusion proteins were obtained using I-TASSER, Q-Mean, ProSA, and Verify3D, while their physicochemical properties, toxicity, and antigenicity were predicted using ProtParam server, ToxinPred server, and VaxiJen server [51].

### 2.2. Linkers for Fusion Proteins

Following translation, protein sequences fold independently into their unique three-dimensional structures. Protein fusion often preserves protein structure while improving stability by reducing protease access and limiting mobility. However, fusion proteins frequently exhibit reduced flexibility due to steric hindrance, which can diminish their biological activity. Consequently, linker peptide sequences are often introduced between proteins when designing fusion proteins [25].

**Figure 4:**
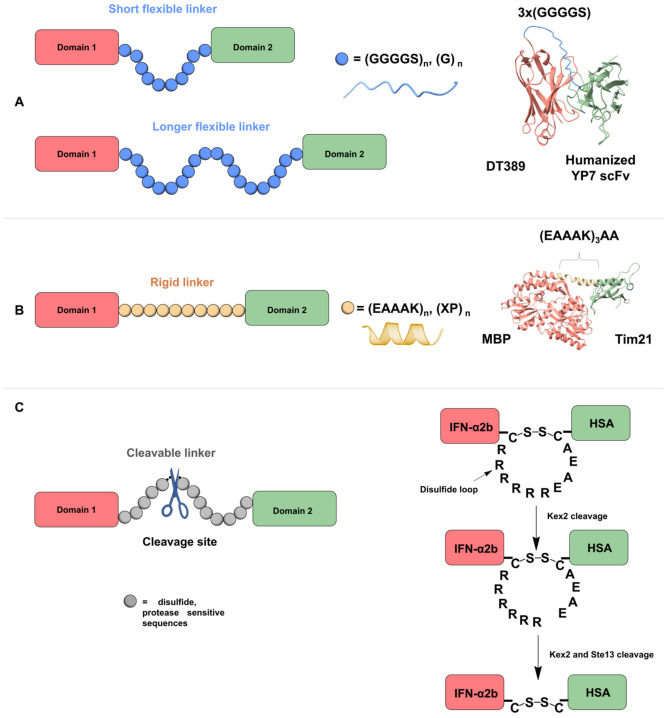
Schematic representation of linker types used for creating fusion proteins. (**A**) Flexible linker (shown in blue). In the DT389–YP7scFv fusion protein, a flexible linker with the sequence (GGGGS)_3_ is placed between the diphtheria toxin fragment DT389 and the YP7-scFv domains, as described in the article [52]. The predicted monomer structure of the DT389–YP7scFv fusion protein was generated using AlphaFold-3 (ipTM = 0.91). (**B**) Rigid linker (shown in yellow). The rigid linker (EAAAK)_3_AA is shown on the right, placed between MBP and Tim21 domains in the MBP–Tim21 fusion protein (PDB: 6K7E; [53]). (**C**) Cleavable linker (shown in gray). On the right, a cleavable linker is placed between the IFN-α2β and human serum albumin (HSA) domains in the IFN-α2β–HSA fusion protein, as described in the article [54]. The predicted monomer structure of the IFN-α2β–HSA fusion protein was generated using AlphaFold-3 (ipTM = 0.77).

Linkers are short peptide sequences of natural or synthetic origin. Notable natural examples include O-glycosylated linkers from cellulases and xylanases, which are rich in proline and hydroxyproline, offering protection against proteolysis [28]. Several types of linkers can be distinguished, including rigid or flexible and cleavable or non-cleavable (see Figure 4).

The flexibility of a linker is influenced by its composition. Flexible linkers typically contain multiple glycine residues, which enable them to form disordered loops [24]. They may also contain serine, threonine, alanine, lysine, and glutamic acid to improve solubility [55]. Including alanine and lysine increases resistance to proteolysis and results in better domain separation within the fusion protein. Some of the most common flexible linkers are the (Gly)n and (Gly-Gly-Gly-Gly-Ser)_n_ sequences. These linkers effectively separate fusion partners, aiding folding and solubility [24]; however, highly homologous repeats in their corresponding DNA sequences can sometimes reduce expression levels and lead to a loss of biological activity. For instance, the ability of a fusion protein containing Vargula luciferase to bind immunoglobulin G (IgG) was lost when a flexible linker was introduced [55]. Rigid linkers, such as the helical (EAAAK)n or proline-rich (XP)n sequences (where X represents any amino acid), are preferred for superior domain separation [28]. Some studies have shown that choosing rigid peptide linkers can lead to improved activity [24]. For instance, insertion of a rigid linker into CAR-T cells containing a rituximab domain resulted in a substantial reduction in tumor growth in vivo and doubled the median survival period compared with the original CAR construct [56].

However, the use of rigid linkers can create steric hindrance between functional domains, thereby reducing their activity. To address this issue, cleavable linkers are introduced between domains in fusion proteins to release the functional domains in vivo and preserve their activity. These linkers can be cleaved in the presence of reducing agents or proteases, such as enterokinase, thrombin, and cysteine proteases. There are two types of cleavable linkers: one containing an intramolecular disulfide bond formed between two cysteine residues and another featuring a thrombin-sensitive sequence (PRS) placed between two cysteine residues [55,57].

Another important parameter of a linker is its length. This affects the separation of fusion protein subunits and influences their expression. Generally, proteins with longer linkers tend to be more active. Examples include the onconase-albumin fusion protein, Fynomer Fc fusion proteins, the human serum albumin (HSA)-interferon α2b fusion protein, and the BH3 domain of the death agonist (BID)-tumor necrosis factor-related apoptosis-inducing ligand (TRAIL) fusion protein [58].

Several bioinformatics tools and databases have been developed for linker selection and design, including LinkerDB (http://www.ibi.vu.nl/programs/linkerdbwww/, accessed 24 April 2026). Key parameters considered in linker design include length, composition, structure, susceptibility to protease cleavage, and interactions with the domains of the fusion protein [28].

## 3. Types of Fusion Proteins

**Figure 5:**
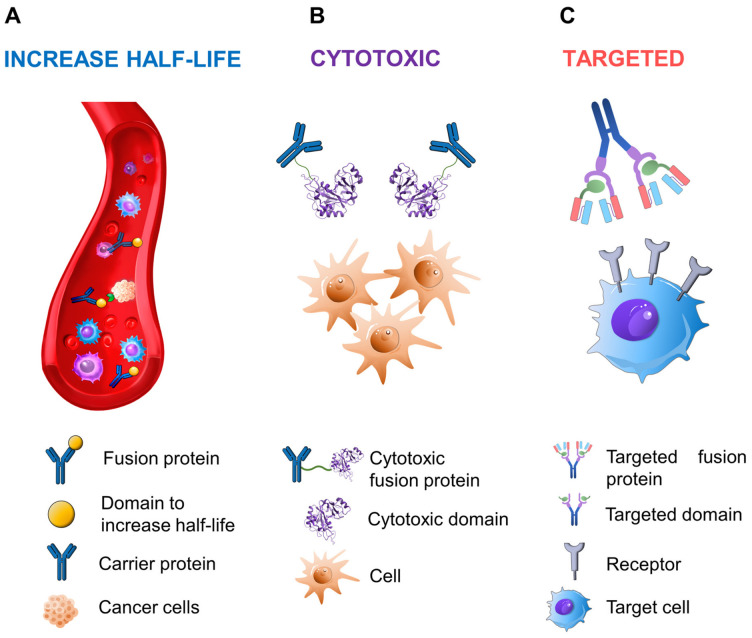
Schematic representation of functional categories of fusion proteins. (**A**) Half-life-extending fusion proteins. Therapeutic proteins with a short half-life (proteins smaller than approximately 70 kDa) are fused to or chemically conjugated with antibody derivatives, albumin, or transferrin to prolong their plasma half-life and preserve their activity. (**B**) Cytotoxic fusion proteins. Cytotoxicity arises from fusing protein effector domains to toxins, immunokinases, or enzymes. (**C**) Targeted fusion proteins for specific cells, organs, and tissues. These enable localized therapeutic effects on target cells, with a targeting domain (typically an antibody) directing the effector domain (for example, cytokines, toxins, or other proteins) to its site of action. Adapted from [26,59,60,61].

**Figure 6:**
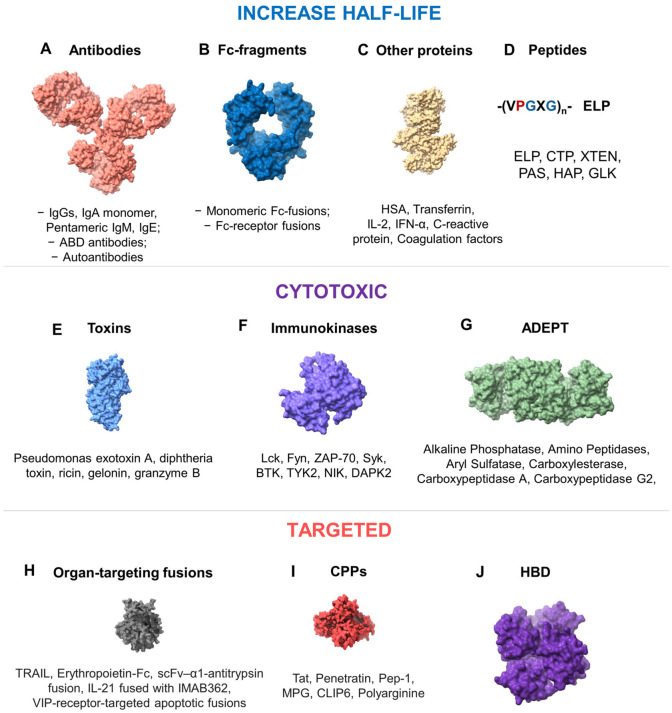
Detailed classification of protein partner types for creating fusion proteins. Half-life extension: (**A**) antibodies; (**B**) Fc fragments; (**C**) other proteins; (**D**) peptides. Cytotoxic: (**E**) toxins; (**F**) immunokinases; (**G**) antibody-directed enzyme prodrug therapy (ADEPT). Targeted: (**H**) organ-targeted fusion; (**I**) cell-penetrating peptides (CPPs); (**J**) heparin-binding domain (HBD). Representative structures are shown: IgG1 (PDB: 1IGY); Fc fragment (PDB: 7LUS); human serum albumin (HSA; PDB: 7DJN); *Pseudomonas* exotoxin A (PDB: 1IKQ); ZAP-70 kinase (PDB: 1U59); ADEPT enzyme (aminopeptidase; PDB: 4ICR); CPP LK-3 (PDB: 9IZ6); and HBD (PDB: 1BHT).

Fusion proteins can be broadly classified into four functional categories: (1) half-life-extending fusion proteins; (2) cytotoxic fusion proteins; (3) targeting fusion proteins; and (4) multifunctional constructs that combine several of these properties [26,59]. These strategies are summarized in Figure 5 and further categorized in Figure 6.

To increase the half-life of a therapeutic protein, fragments of the crystallizable fragment (Fc) of immunoglobulins (Figure 6B), albumin (Figure 6C), or transferrin are commonly used (Figure 6). Polypeptide chains (Figure 6D) can also serve as fusion partners for this purpose, including proline-, alanine-, and serine-based polypeptides (PASylation); alanine-, glutamic acid-, glycine-, proline-, serine-, and threonine-based polypeptides (XTEN); and glycine-based polypeptides (HAP). More recently, flexible peptides such as ELPs and the C-termini peptides (CTP) have been used to create fusion proteins that avoid renal clearance. Compared with PEGylated proteins, these constructs enable peptides of defined lengths and properties to be designed while also achieving higher yields of the target protein [19].

Numerous drugs based on fusion proteins containing the crystallizable fragment (Fc) have been approved for half-life extension, including etanercept, abatacept, aflibercept, rilonacept, romiplostim, belatacept, dulaglutide, asfotase alfa, luspatercept-aamt, and follitropin beta [26]. These fusion proteins combine an antibody with another biologically active protein domain or peptide to prolong the half-life [62]. Human serum albumin (HSA) has been used much less frequently to produce fusion proteins. To date, only one such drug, albiglutide (Tanzeum^®^), has been approved [49]. A fusion protein based on HSA and interferon alpha-2b was recently tested in a phase 1/2 clinical trial involving patients with hepatitis B [63]. Among peptides, ELP is the most frequently used fusion partner in clinical trials. Several Phase 1 and Phase 2 studies have been conducted using ELP-based fusion proteins, including PB1023 and PE0139 for treating diabetes and PB1046 for treating cardiopulmonary disease [61].

Cytotoxic fusion proteins (Figure 6) utilize immunotoxins (E), which are derived from bacterial or plant toxins. These fusion proteins are usually categorized as either cell-surface fusion proteins, which activate immune cells, or fusion proteins that become active only after entering the cell [59]. Examples of toxins used as cytotoxic components include *Pseudomonas* exotoxin A (PEA), diphtheria toxin, ricin, gelonin, granzyme B, and RNases. Fusion proteins based on these toxins typically comprise three domains: a cell-binding domain; a domain that transports the toxin into the target cell; and a catalytic domain that induces cell death. Saporin has been linked to numerous monoclonal antibodies to create first-generation immunotoxins that target leukemia and lymphoma cells [64]. The catalytic domains of PEA or diphtheria toxin, together with natural ligands such as TGF-α or EGF, have been used to target the EGF receptor in tumors [65]. One notable example of a recently developed cytotoxic fusion protein is based on the serine protease granzyme B and a humanized anti-HER2 scFv. This construct has demonstrated high in vivo efficacy and a long half-life of 39.2 h [66]. Clinically relevant examples include Denileukin diftitox and Tagraxofusp-erzs [26].

Unlike immunotoxins, the use of immunokinases (Figure 6F) enable the targeted destruction of tumor cells without harming normal cells [67]. In one study, the immunokinase serine/threonine-associated protein kinase (DAPK1) was used to develop an antileukemic fusion protein that targets CD22 on neoplastic B cells [68].

In the antibody-directed enzyme prodrug therapy (ADEPT) strategy (Figure 6G), an antibody is linked to an exogenous enzyme. The prodrug is then administered into the bloodstream, where the enzyme, which has been pre-localized to the tumor site, converts it into a potent cytotoxic agent [69]. For example, a Phase 1 clinical trial of ADEPT employed a combination of the F(ab′)2 fragment of a mouse monoclonal antibody against CEA (A5B7) and bacterial carboxypeptidase (CPG2) to deliver a benzoic acid-mustard prodrug to leukemia cells [70]. However, clinical studies of immunokinase-based fusion proteins and the ADEPT strategy have remained limited to date.

Targeting fusion proteins (Figure 6) combine a binding domain, often derived from antibodies, with a therapeutic effector such as a cytokine, an enzyme, a signaling molecule, or a genome-editing component (e.g., Cas9) [26]. One of the recently approved Fc-fusion targeting proteins is Strensiq^®^, a tissue-specific alkaline phosphatase used to treat hypophosphatasia [58]. Another recently approved fusion protein in this class for the treatment of metastatic uveal melanoma is Tebentafusp-tebn. This bispecific antibody targets the gp100 peptide and is fused to a single-chain variable fragment of anti-CD3 [71]. Noteworthy recent developments include a fusion protein based on murine interleukin-21 (IL-21) fused to the C-termini domain of IMAB362 (an antibody-based clinical drug targeting Claudin 18.2), which demonstrated a more pronounced antitumor effect in colon adenocarcinoma than either IMAB362 or IL-21 alone [72]. One of the targeting components used to produce fusion proteins for cancer therapy is TRAIL. TRAIL triggers apoptosis in tumor cells by activating corresponding cell surface receptors and has minimal effects on normal cells (Figure 6H) [73]. Examples of targeting components used to produce fusion proteins for cancer therapy include antibodies (e.g., CD19 IgG1), antibody fragments (such as the 4D5 single-chain variable fragment of anti-HER2), and peptides (e.g., the RGR peptide) that have been used as fusion partners with TRAIL. However, the number of clinical studies of TRAIL-based fusion proteins remains limited [74].

CPPs (see Figure 6I), including TAT and penetratin, are among the earliest examples of targeting domains that enable intracellular delivery [75]. A key advantage of using CPPs as fusion partners is their ability to effectively enter cells in a non-invasive manner, thereby enhancing the efficiency of endosomal escape [76]. The HBD exhibits high affinity for cells that express heparan sulfates on their surface, such as tumor cells that overexpress HER2/3/4 receptors [77]. Currently, clinical trial data for HBD-based fusion proteins remain limited.

These previously established fusion strategies, which were initially developed for other therapeutic proteins, have subsequently been adapted for L-ASNases (as described in Section 4) to overcome their specific pharmacological limitations, including short half-life, immunogenicity, and limited tumor selectivity.

## 4. Fusion Partners for L-ASNase

As with other bacterial proteins, L-ASNase has a short half-life in serum and is rapidly eliminated via renal filtration. This issue is typically addressed through protein modification [78]. The main strategies for modifying L-ASNase involve immobilization on various carriers [79] or the use of fusion technology. In the latter approach, L-ASNase is fused to proteins or domains with a longer half-life, such as albumin, Fc fragments, ELPs, or XTEN [80]. Specific cell surface proteins on tumor cells can also serve as targets for antibody–drug conjugates.

### 4.1. Tags for Improving the Solubility and Expression of L-ASNase

**Figure 7:**
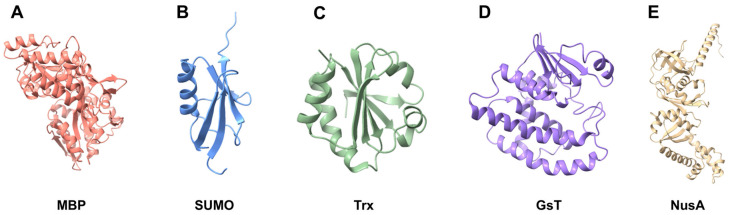
Tags used to improve the solubility and expression of L-ASNase. (**A**) MBP from *E. coli* (PDB: 4MY2). (**B**) Ubiquitin-like protein SMT3 (SUMO) from *S. cerevisiae* (PDB: 1EUV). (**C**) Thioredoxin (Trx) from *E. coli* (PDB: 2TRX). (**D**) Glutathione S-transferase (GST) from *S. japonicum* (PDB: 1UA5). (**E**) N-utilizing protein A (NusA) from *E. coli* (PDB: 5LM9).

To facilitate purification, many L-ASNases are expressed with a hexahistidine (His) tag at the end [81]. In addition to this tag, affinity tags such as the FLAG and the Strep II tags are also used. The main disadvantage of using these tags is that they can affect the expression level and solubility of L-ASNases. To overcome this limitation, a combination of a solubility-enhancing tag and an affinity tag is typically employed [82]. Tags that increase the solubility of L-ASNases (Figure 7) include MBP, SUMO [83], the N-utilizing protein A (NusA), thioredoxin (Trx), and glutathione S-transferase (GST) [84,85]. Trx is a small redox-related protein that has been used as a fusion tag or protein chaperone to increase soluble expression. It has been suggested that this redox function plays a role in increasing the expression of recombinant human proteins in *E. coli*. MBP is relatively large among fusion partners, with a molecular weight of approximately 42 kDa. It enhances the solubility of target proteins and can be easily purified using amylose resin. The SUMO tag, derived from the SUMO protease, has been shown to enhance soluble expression, particularly for proteins that are difficult to express. GST is a monomeric protein of approximately 26 kDa that forms a homodimer. It is a classic fusion format for soluble expression and for glutathione-affinity purification or pull-down assays of recombinant proteins [86]. We previously discussed examples of L-ASNases with these tags in detail in our review [85].

### 4.2. Fusion Proteins for Half-Life Extension of L-Asparaginase and Immunogenicity Reduction

Unlike traditional chemotherapy, targeted cancer therapy selectively acts on the key tumor cell molecules responsible for growth and progression while reducing toxicity to healthy tissues [87]. Various protein-based drugs are used for targeted therapy, with antibodies being the most common [19].

#### 4.2.1. Antibody Fusion Proteins

**Figure 8:**
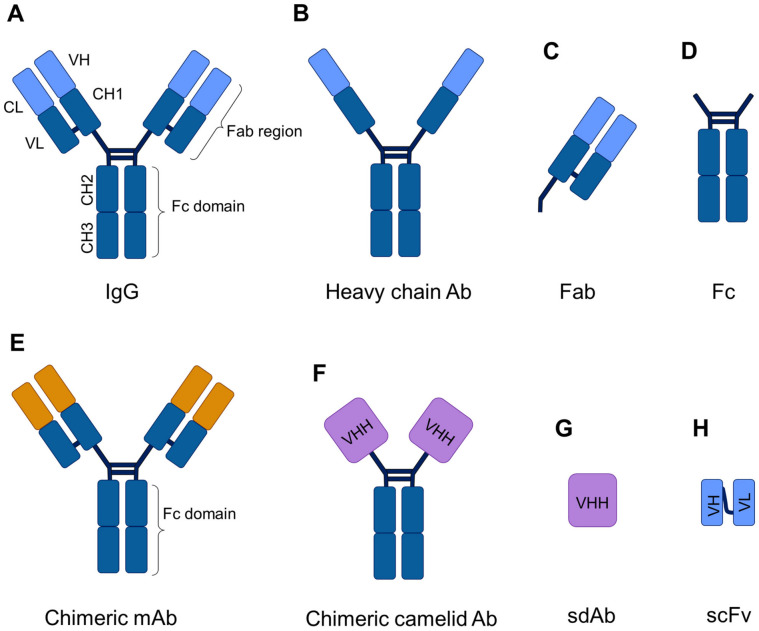
Main types of antibodies used in fusion proteins. (**A**) IgG. The complete IgG molecule is Y-shaped and consists of two heavy chains and two light chains linked by disulfide bonds. It has two Fab arms for antigen binding and an Fc region for effector functions. (**B**) Heavy chain antibody (Ab). A heavy-chain-only antibody derived from camelids (e.g., llamas or camels) that lacks light chains. Each heavy chain contains a single antigen-binding domain (VHH), forming a homodimer. (**C**) Fab. The antigen-binding fragment of an IgG, comprising one light chain and the VH-CH1 portion of the heavy chain. (**D**) Fc. The crystallizable fragment derived from the IgG Fc region (CH2-CH3 domains). (**E**) Chimeric monoclonal antibody (mAb). A monoclonal antibody that combines variable regions from a non-human source (e.g., a mouse) with human constant regions. (**F**) Chimeric camelid antibody (Ab). A hybrid antibody created by grafting a camelid VHH domain onto a human IgG. (**G**) sdAb (single-domain antibody). Also known as a nanobody (VHH from camelids), this is the smallest functional antigen-binding unit. (**H**) scFv. A fusion of the heavy (VH) and light (VL) variable domains connected by a flexible peptide linker (10–25 amino acids) [90].

The diversity of antibody fusion proteins has increased through the attachment of various payloads to different antibody components, including full-length IgGs, Fc domains, scFvs, single-domain antibodies (sdAbs), nanobodies, and antigen-binding fragments (Fabs), among others (Figure 8) [88]. To extend the half-life of drugs, antibodies in fusion proteins can be used in full IgG form or as antibody fragments for faster clearance from the blood and local action [89]. Monoclonal antibodies, scFvs, and nanobodies have been developed for fusion with L-ASNases, as described below.

##### Monoclonal Antibody

One of the earliest L-ASNase fusion proteins was developed in 1992. This protein paired the enzyme with a non-inhibitory monoclonal antibody (mAb) in order to protect it from proteolytic inactivation in plasma. This protein lacked a linker and consisted of affinity-bound proteins. The mAbs were isolated from the spleens of BALB/c mice three days after immunization with L-ASNase EcA II. Of the six candidates screened, the researchers selected the one with the highest affinity. Among these proteins, mAb#12-4 provided approximately 72% protection against trypsin inactivation at the Lys29 site [31].

##### Anti-L-Asparaginase Single-Chain Antibody

ScFvs have been used as protein partners for L-ASNase to produce fusion proteins and increase the enzyme’s half-life. In a follow-up study by the same authors, the L-ASNase gene was co-expressed with the scFv gene of mAb#12-4. As in their previous work, the aim was to fuse L-ASNase with the antibody to enhance its resistance to proteolytic degradation. The N-terminus of the fusion protein contained a His_6_ tag, followed by the antibody sequence. A (Gly_4_Ser)_3_ linker connected the antibody’s light and heavy chains, while a 2 × (Gly_4_Ser)_3_ linker connected the scFv to the L-ASNase sequence lacking a signal peptide. The fusion protein had a molecular weight of 65,127 Da and a specific activity of 13.3 IU/mg and was resistant to inactivation by trypsin at concentrations of 125–1000 IU/mL [91].

To increase the resistance of L-ASNase from EcA II to proteolysis, Guo et al. also developed a fusion protein comprising L-ASNase and the scFv46 antibody [92]. The L-ASNase EcA II sequence was placed at the N-terminus of the first fusion protein and at the C-terminus of the second. A long (Gly_4_Ser)_6_ linker was designed using the Insight II package. The protein model was initially evaluated using Insight II’s Homology and Discover modules to remove steric hindrances, generate a loop region, and model the fusion protein tetramer. Fusion of scFv to the N-terminus of L-ASNase was shown not to alter the interaction between ASNase subunits. Compared with native ASNase, the scFv-ASNase and ASNase-scFv hybrid proteins were more resistant to treatment with proteolytic enzymes, retaining 94% and 70.3% of their initial activity, respectively. Additionally, the in vitro half-life of the fusion L-ASNases increased to 9 and 6 h, respectively, whereas the half-life of the native L-ASNase was 2 h. Meanwhile, the specific activity (102 U/mg) and K_m_ (6.34 × 10^−5^ M) of scFv-ASNase were comparable to those of the native enzyme. However, the specific activity of ASNase-scFv decreased sixfold, and its K_m_ increased twofold [93].

##### Nanobody

One type of antibody derivative used to create fusion proteins is the nanobody. Nanobodies, also known as variable heavy chain antibodies (VHHs), are single variable domains from the heavy chains of antibodies found in llamas and other camelids. Unlike monoclonal antibodies, nanobodies have a small molecular weight of 15 kDa and consist of antigen-binding sites within hypervariable regions (complementarity-determining regions, or CDRs). The longer sequences found in the CDR3 loop enable nanobodies to interact more effectively with antigens than conventional antibodies [30,94].

**Figure 9:**
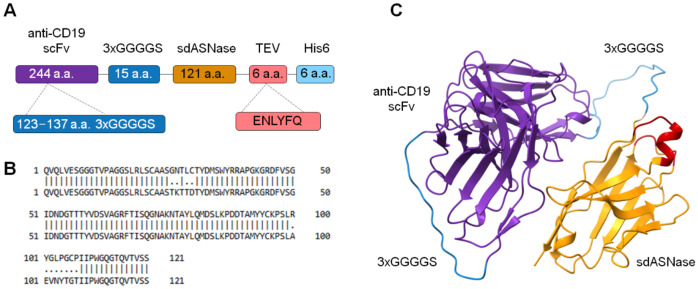
Schematic structure of the anti-CD19–EcA II fusion protein. (**A**) Genetic construct of the anti-CD19 scFv antibody fusion protein derived from a camelid sdAb and EcA II-derived sdASNase. The sequence of the anti-CD19 scFv was taken from PCT/IB2018/057037. The figure does not show the glycine residue after the second linker. (**B**) Pairwise sequence alignment between the VHH domain of a camelid sdAb (top; PDB: 1KXV) and sdASNase (bottom), obtained using EMBOSS MATCHER (https://www.ebi.ac.uk/jdispatcher/psa, accessed 10 April 2026). (**C**) Predicted monomer structure of the anti-CD19 sdASNase fusion protein, generated using AlphaFold 3 with an ipTM of 0.64. The amino acid residues taken from L-ASNase EcA II are shown in red. Structural alignment in PyMOL 3.1 (accessed 10 April 2026) demonstrates high similarity: the RMSD is 0.711 Å between the camelid VHH domain (PDB: 1KXV) and the AlphaFold 3 fusion protein model.

A truncated form of L-ASNase was fused with an anti-CD19 scFv antibody (FMC63 clone) [83] to create a fusion protein termed “Targeted Catalytic Nanobody” (T-CAN) (Figure 9). 

**Figure 10:**
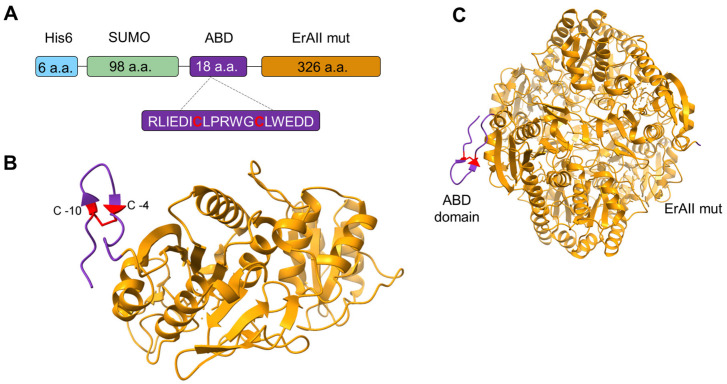
Schematic structure of the ABD-ErA II fusion protein. (**A**) Genetic construct of the ABD-ErA II fusion protein. (**B**) Structure of tetrameric fusion protein (PDB: 7U6M). Only one of the four ABDs is shown. (**C**) The ABD peptide sequence contains two cysteines (shown in red), which form a disulfide bridge (also shown in red).

A camelid nanobody was used as a template to generate a truncated protein with asparaginase activity (sdASNase) [95]. The key catalytic residues of L-ASNase EcA II (Thr12, Lys162, Asp90, Thr89, Tyr25, and Glu283), along with their adjacent amino acids, were transferred to the single-domain antibody (sdAb) template (a total of 12 amino acids in total were taken from the EcA II sequence). Several other amino acid substitutions were also introduced into the sdAb sequence. The T-CAN fusion protein includes the scFv antibody fragment that targets CD19, a 3 × G_4_S linker that connects the scFv fragment to sdASNase, and a sequence that encodes the tobacco etch virus protease recognition site (TEV, amino acids ENLYFQ). A His_6_ tag was added at the 3′ end (Figure 10). The molecular masses of sdASNase and T-CAN were 14.9 kDa and 42 kDa, respectively, compared with 135 kDa for EcA II. The specific activity of the purified T-CAN fusion protein was higher than that of sdASNase (4.30 ± 0.26 U/mg versus 1.09 ± 0.29 U/mg). The IC_50_ values against MOLT-4 cells for sdASNase and T-CAN were higher than that of wild-type L-ASNase (0.13 ± 0.02 U/mL and 0.12 ± 0.01 U/mL, respectively).

#### 4.2.2. Albumin-Binding Domain

In two studies, short-chain ABDs were used as fusion partners for L-ASNase to extend the enzyme’s half-life. In the study by Van Trimpont et al., a fusion protein comprising an ABD at the N-terminus and ErA II at the C-terminus (PDB ID: 7U6M) was developed (see Figure 10).

The 18-amino acid peptide SA21, which binds most strongly to HSA (467 nM), was used as the ABD. The L-ASNase sequence contained three mutations near the active site (A31I/E63Q/S254Q). Following the introduction of these mutations into ErA II, the enzyme’s K_m_ for asparagine increased from approximately 50 mM to approximately 100 mM. After fusion with the ABD, this value increased further to approximately 150 mM. The specific activity of the fused enzyme was comparable to that of ErA II without tags (417.7 ± 13.6 vs. 443.5 ± 4.8 IU/mg, respectively). However, the fusion protein had a longer half-life (38.3 h) than the free form of the enzyme (2.3 h) and ErA II fused to a SUMO tag (15.7 h). An in vivo study revealed comparable long-term anti-leukemia inhibitory effects with PEGylated L-ASNase and L-ASNase fused to the ABD. Both PEGylated ErA II and ABD-ErA II retained enzymatic activity, significantly reducing blood asparagine levels to below 10 μM for 43 days. However, the fusion protein exhibited fewer immune-related side effects [96].

Patent WO2025026424 describes the engineering of a fusion protein involving the linking of the 46-amino acid ABD-3 domain to L-ASNase ErA II via a (GGGGS)_5_ linker. Binding of the ABD-3 domain of the fusion protein to albumin has been shown to result in the formation of a substantial complex. This complex can evade renal clearance and resist the effects of antibodies, leading to a significant increase in half-life. Compared with native L-ASNase ErA II, the fusion protein exhibited slightly reduced specific activity (399.7 IU/mg versus 482.6 IU/mg). However, its substrate affinities remained almost identical, with K_m_ values of 0.14 mM (versus 0.11 mM) for asparagine and 1.13 mM (versus 0.91 mM) for glutamine. The resulting ABD-ErS5 fusion protein induced caspase-dependent apoptotic cell death in the MKN-45 human gastric cancer cell line. Although tumor cell survival rates were similar for the fusion protein and native L-ASNase ErA II, the half-life of the fusion protein in mouse serum increased 18-fold (6.83 ± 0.13 days versus 0.40 ± 0.01 days) [97].

Therefore, ABD-based fusion is one of the most effective approaches currently reported for extending the half-life of L-ASNase while maintaining enzymatic activity.

#### 4.2.3. Elastin-like Peptide

Due to its biocompatibility, low immunogenicity, and ability to prolong half-life, ELP is currently widely used to create therapeutic fusion proteins for delivery to various targets. ELP is a synthetic peptide consisting of a valine-proline-glycine-X-glycine motif (VPGXG)n, where X can be any amino acid except proline and n is the number of repeats [98]. Previous studies have shown that fusing ELPs with small therapeutic proteins increases the half-life of the therapeutic proteins by creating a larger hydrodynamic radius, which in turn avoids rapid renal clearance [61]. Wang et al. demonstrated that ELP consisting of VPGVG repeats increases the half-life of interferon-alpha and reduces the likelihood of side effects [99].

The authors therefore designed this peptide to create another fusion protein with L-ASNase EcA II, aiming to reduce its immunogenicity. Using AlphaFold2, they first identified the most promising ELP lengths within the fusion proteins: ELP60 (VPGVG)_60_ and ELP90 (VPGVG)_90_. These proteins have a core composed of an L-ASNase tetramer surrounded by an ELP shell, and the long ELP chains can reduce the accessibility of immunogenic epitopes of L-ASNase. The resulting fusion proteins exhibited activity comparable to that of native L-ASNase and greater than that of PEGylated L-ASNase. Furthermore, compared with native L-ASNase, the fusion protein retained activity for 2.5 times longer during long-term storage (25 days versus 10 days).

Additionally, fusing L-ASNase with ELP60 or ELP90 increased the protein’s hydrodynamic size. The fusion proteins were approximately 2.2- and 2.7-fold larger than native L-ASNase, with hydrodynamic diameters of 18.6 and 22.5 nm and molecular masses of 60 and 70 kDa, respectively. This is in contrast to the dimensions of 8.4 nm and 36 kDa observed for native L-ASNase [100]. These parameters can contribute to the increased half-life of L-ASNase-ELP by decreasing renal clearance, because the threshold peptide size for renal filtration is known to be less than 70 kDa [101].

The fusion protein formed a depot at the injection site in mice, from which it was gradually released. For L-ASNase-ELP90, the release time was 4.1-fold longer. Peak concentrations of the fusion proteins were threefold and fourfold lower than those of native EcA II (for ELP60 and ELP90, respectively). Therefore, these fusion proteins caused no side effects, except for elevated AST and ALT in the L-ASNase-ELP60 group. L-ASNase-ELP90 exhibited the lowest immunogenicity and had the longest half-life (353.2 h), compared with L-ASNase-ELP60 (191 h) and native L-ASNase EcA II (3 h). Administration of the L-ASNase-ELP90 fusion protein in mouse leukemia models increased survival by 1.25-fold and 2.12-fold, respectively, compared with L-ASNase-ELP60 and native L-ASNase. Similar results were obtained in a mouse lymphoma model [100].

In another study, the same authors combined ASNase-ELP90 with an anti-programmed cell death protein-1 (aPD-1) immune checkpoint inhibitor antibody to enhance cytotoxicity against solid tumors. Compared with the native enzyme, the fusion protein exhibited enhanced in vitro and in vivo cytotoxicity against oral cancer, melanoma, and cervical cancer, with no detectable systemic toxicity. A study investigating the long-term efficacy of L-ASNase-ELP90 in conjunction with an immune checkpoint inhibitor for treating metastatic melanoma revealed that L-ASNase-ELP90 was ineffective at preventing metastasis in the long term. Tumor metastases were observed in this group of mice by day 60 after administration, and the median survival time was 70 days. When combined with aPD-1, the survival time increased to 80 days, whereas a PD-1 monotherapy resulted in a shorter survival time of 31 days [102].

### 4.3. Cytotoxic Fusion Proteins

#### 4.3.1. Cholesteryl Ester Transfer Protein

**Figure 11:**
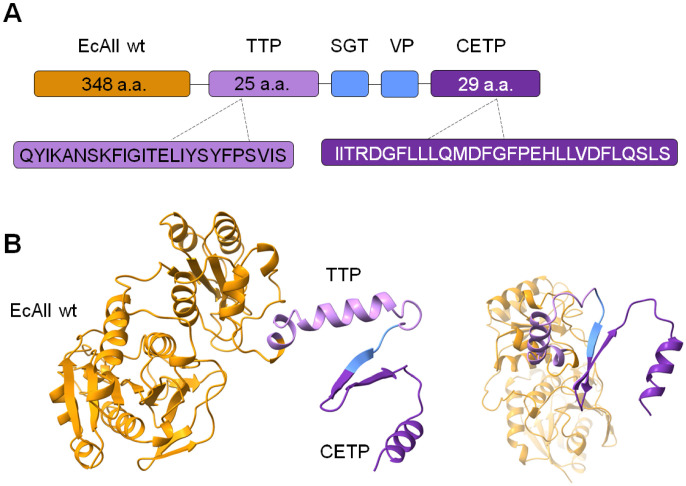
Schematic structure of the EcA II-TTP-CETP fusion protein. (**A**) Genetic construct of the fusion protein comprising L-ASNase EcA II, TTP, and CETP. The SGT peptide was used as a linker between TTP and CETP. No role is specified for the VP amino acid residues. (**B**) Predicted monomer structure of the EcA II-TTP-CETP fusion protein, generated using AlphaFold 3 with an ipTM of 0.81.

Some studies have involved engineered L-ASNase-based fusion proteins to enhance immune responses. One such protein is L-ASNase EcA II (WP_033810809.1), which includes a tetanus toxin helper T-cell epitope (TTP, QYIKANSKFIGITELIYSYFPSVI), a Ser-Gly-Thr (SGT) linker, and cholesteryl ester transfer protein (CETP) residues (see Figure 11). This protein has been used as a prototype for a vaccine against atherosclerosis.

The fusion protein contains several CETP-derived epitopes that are presented to antigen-presenting cells. L-ASNase acts as a carrier, while TTP increases the antigenicity of the CETP epitope. CETP is involved in the transfer of cholesteryl esters from high-density lipoproteins (HDL) to low- and very-low-density lipoproteins (LDL and VLDL). The accumulation of these lipoproteins leads to atherosclerosis. Inhibiting CETP activity is therefore a promising strategy for reducing the risk of this disease. However, the human B-cell epitope antigen against CETP has only a weak inhibitory effect on CETP, so additional components were incorporated into the fusion protein to enhance its function. The activity of L-ASNase-TTP-CETP was approximately 83% of that of native L-ASNase EcA II (200 U/mg versus 240 U/mg). In mice, three doses elicited a robust anti-CETP immune response, with antibody titers reaching 1:12,800 and persisting in serum for over 18 weeks. Despite the strong CETP-specific immune response, no subsequent pathological changes were detected in the organs of the mice [103]. Overall, fusion proteins that combine L-ASNase with immunostimulatory domains show promise as vaccine platforms, as they enable the localized induction of robust immune responses in vivo. However, further studies are needed to establish their safety profile and applicability beyond experimental immunization models.

#### 4.3.2. Arginase

Another study aimed to increase the cytotoxicity of a multimeric enzyme based on the hybrid L-ASNase 63N-hC sequence (derived from guinea pig and human L-ASNase), as described in reference [104], as well as human arginase I (ARG1, UniProt P05089). The N-termini sequences of L-ASNase (63N-hC) and ARG1 were linked by a rigid (EAAAK)_2_A linker, resulting in a 905-residue monomer. The structure of the 63N-hC-linker-ARG1 monomer was modeled using AlphaFold2 and validated using MolProbity to assess the quality of the stereochemistry. Despite successful refolding and purification, the activity of the fusion protein was found to be 4.7 ± 0.9 IU/L [105]. Further development of the technology for fusing chimeric L-ASNase with arginase, as well as for fusions with antibody fragments, is therefore required.

### 4.4. Fusion Proteins Targeted to Specific Cells, Organs and Tissues

One promising therapeutic approach for targeting tumor cells involves the creation of fusion proteins that include targeted fusions, CPPs, and peptides or proteins with antitumor activity [50].

#### 4.4.1. Anti-CD19 Single-Chain Antibody

**Figure 12:**
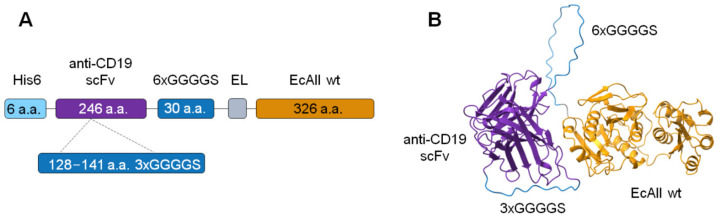
Schematic structure of the anti-CD19 scFv-EcA II fusion protein. (**A**) Genetic construct of the fusion protein comprising the anti-CD19 scFv antibody fused to wild-type L-ASNase EcA II. (**B**) Predicted monomer structure of the anti-CD19 scFv-EcA II fusion protein generated using AlphaFold 3 with an ipTM of 0.59. Structural alignment in PyMOL shows a high degree of similarity: the RMSD is 0.431 Å between L-ASNase EcA II (PDB: 3ECA) and the AlphaFold 3 fusion protein model, and 0.565 Å between the FMC63 scFv (PDB: 7URV) and the AlphaFold 3 fusion protein model.

To create a fusion protein with L-ASNase, the human CD19 surface receptor (hCD19), which is expressed on the surface of ALL cells, was selected. The genetic construct (see Figure 12) included an N-termini His_6_ tag, a sequence encoding an anti-CD19 antibody fragment (FMC63 scFv) [106], six repeats of the GGGGS linker sequence, and wild-type L-ASNase EcA II. The activity of the fusion protein was found to be 90.9 ± 12.9 U/mg. Wild-type EcA II and the fusion protein exhibited similar IC_50_ values: 0.002 and 0.004 U/mL for RS4 cells and 0.48 and 0.50 U/mL for Raji cells, respectively. The fusion protein exhibited dose-dependent binding to CD19 [107].

#### 4.4.2. Cell-Penetrating Peptides and Proteins

**Figure 13:**
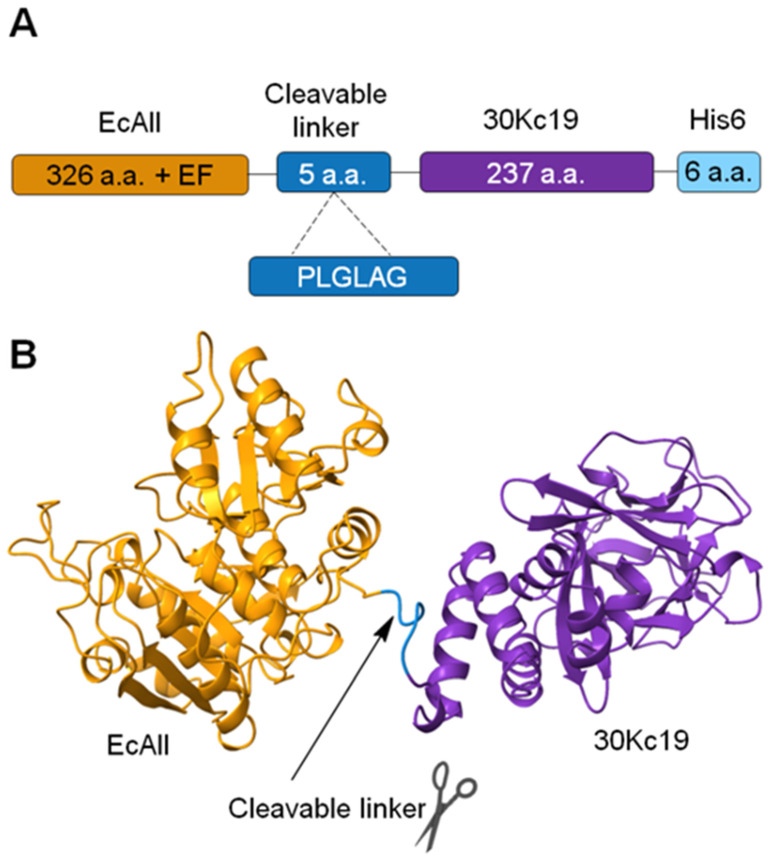
Schematic structure of the EcA II-30Kc19 fusion protein. (**A**) Genetic construct of the L-ASNase EcA II-30Kc19 fusion protein. (**B**) Predicted monomer structure of the EcA II-30Kc19 fusion protein, generated using AlphaFold 3 with an ipTM of 0.63. Structural alignment in PyMOL shows a high degree of similarity: the RMSD is 0.237 Å between the EcA II domain (PDB: 3ECA) and the AlphaFold 3 fusion protein model.

CPPs are cationic and/or amphipathic peptides that can penetrate cells without involving membrane receptors. They usually have low cytotoxicity. They are used to deliver bioactive substances and large molecules, which can be attached either covalently or noncovalently [108]. Protein 30Kc19 (NP_001095197, 254 a.a.), which is found in the hemolymph of the silkworm *Bombyx mori*, can penetrate a variety of cell types effectively [109]. To enhance the half-life and cytotoxicity of L-ASNase EcA II against leukemia cells, researchers developed a fusion protein with L-ASNase at the N-terminus and CPP 30Kc19 at the C-terminus (see Figure 13). To reduce steric hindrance between these two domains, a cleavable PLGLAG linker, recognized by metalloproteinases, was introduced. This linker was chosen for the targeted delivery of L-ASNase to leukemia cells, which are characterized by high levels of metalloproteinases. The resulting protein was expressed in a soluble form and exhibitied increased activity (11.4 IU/mL versus 7.3 IU/mL) and stability compared with the fusion protein without the linker. The fusion protein with the linker was also able to penetrate leukemia cells and hydrolyze intracellular asparagine, exhibiting increased cytotoxicity and selectivity for mouse lymphoblastic L5178Y cells compared with the fusion protein without the linker and native L-ASNase EcA II [110].

Another study used a CPP with a protein transduction domain (PTD) to generate a fusion protein and deliver L-ASNase intracellularly. Among CPPs, the trans-activating regulatory protein (TAT) from lentiviruses has recently been shown to effectively deliver various covalently linked macromolecules across cell membranes. The transduction mechanism of the positively charged TAT is mediated by its binding to negatively charged heparan sulfate on the cell surface. This binding is inhibited in the presence of heparin and heparan sulfate. As PTD proteins, including TAT, can cause systemic toxicity themselves, the authors fused the TAT peptide with L ASNase, regulating the intracellular uptake of the fusion protein by adding protamine and heparin. The fusion protein was produced using N-hydroxysuccinimide ester of 3-(2-pyridyldithio)propionic acid (SPDP) to chemically cross-link the TAT peptide (CGGGYGRKKRRQRRR) with the L-ASNase EcA II via a disulfide linkage. Once inside the cell, the disulfide bonds between TAT and ASNase are expected to break due to the presence of various reducing agents in the cytosol. The specific activity of TAT-L-ASNase was 60% (124 ± 20 IU/mg) of that of native L-ASNase. TAT-L-ASNase exhibited comparable cytotoxic activity to that of the native enzyme (IC_50_ values of 0.0102 and 0.0100 IU/mL, respectively) against MOLT-4 cell culture. In contrast to FITC-labeled native L-ASNase, FITC-labeled TAT-L-ASNase effectively penetrated various cell lines expressing significant amounts of this protein. The addition of heparin inhibited the penetration of TAT-L-ASNase-FITC, while the addition of protamine counteracted this effect of heparin. A study of the in vivo action of TAT-L-ASNase showed that therapy with this protein increased the average survival time of mice with murine T-lymphoma L5178Y from 13.9 days with native L-ASNase to 15.6 days [111].

#### 4.4.3. Heparin-Binding Domain

Unlike strategies primarily designed to increase circulation time, HBD-mediated fusion provides an additional mechanism by promoting interaction with heparan sulfate-expressing tumor cells, which could potentially improve tumor localization. To enhance the stability and half-life of L-ASNase from *Wolinella succinogenes* (WsA), Sannikova et al. created a genetically engineered fusion protein. The L-ASNase sequence, containing the V23Q and K24T mutations that increase resistance to trypsinolysis, was fused to the N-terminus of the heparin-binding sequence KRKKKGKGLGKKR. This composition was chosen for the L-ASNase fusion protein because heparin-binding domains (HBDs) are found in blood proteins, on the surface of cells, and in the extracellular matrix. Furthermore, WsA L-ASNase exhibits reduced glutaminase activity compared to EcA II and ErA II.

To link the L-ASNase to the HBD, the Pch PRP8 intein gene carrying the C1A (Cys1Ala) and C8Y (Cys8Tyr) mutations was inserted. The resulting fusion L-ASNase (Was79) exhibited asparaginase activity comparable to that of the native L-ASNase Was02 (187 ± 12 versus 238 ± 14 IU/mg), as well as reduced glutaminase activity. Was79 was also bound effectively to the surface of K562 cells through the selective binding of its HBD. In mice with L5178Y lymphadenosis, Was79 demonstrated the highest efficacy of all the tested L-ASNases at all the tested single-dose levels (125–8000 IU/kg). At doses of 500–2000 IU/kg, survival was almost 1.5 times higher than with treatment using the native enzyme (19/21 versus 13/20). Complete remission was achieved in all cases with single doses of 1000 or 2000 IU/kg of Was79 administered five times at 48 h intervals [112].

#### 4.4.4. Tumor Necrosis Factor-Related Apoptosis-Inducing Ligand

**Figure 14:**
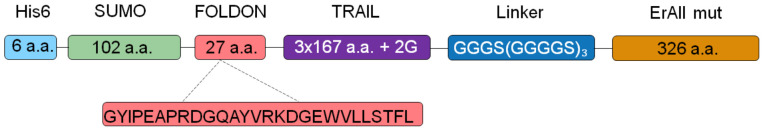
Schematic structure of the SUMO-TRAIL-ErA II fusion protein. Genetic construct schematic of the SUMO-TRAIL-ErA II fusion protein.

To enhance the cytotoxic activity of L-ASNase ErA II (UniProt P06608; A31I, E63Q, and S254Q mutations), a fusion protein was created by attaching three tandem repeats of the soluble domain of human TRAIL (UniProt P50591, residues 115–281). This fusion protein (Figure 14) was designed to overcome L-ASNase-induced apoptosis resistance in glioma cells through the TRAIL moiety. Previous studies have demonstrated the synergistic antiproliferative effect of L-ASNase and TRAIL on SF188 and LN229 glioblastoma cells, as well as significant tumor growth inhibition in vivo [113]. To enable efficient trimerization, the FOLDON sequence was added to TRAIL, and a SUMO tag was introduced at the N-terminus of FOLDON. A 19-residue linker, (GGGS(GGGGS))_3_, connects the third TRAIL repeat to the ErA II mutant. The fusion protein exhibited significantly higher cytotoxicity against human acute myeloid leukemia MV4 cells than free L-ASNase ErA (IC_50_ = 0.064 IU/mL vs. 1.631 IU/mL). Compared with free L-ASNase, the TRAIL–ErA fusion protein had a prolonged half-life and also exhibited high cytotoxic activity in immunodeficient mice bearing MV4 tumors [83].

A comparative summary of L-ASNase fusion constructs is presented in Table 2, which summarizes the implementation of these strategies and their current translational status.

A comparative analysis of the L-ASNase fusion strategies currently available indicates that no single approach provides an optimal solution for all pharmacological limitations. Available data suggests that the fusion of L-ASNase with ABD or HBD currently yields the most balanced enhancement of pharmacokinetic properties and therapeutic efficacy. In contrast, ELP-based fusion proteins primarily increase circulation time and reduce immunogenicity. Antibody-derived fragments and CPPs offer additional targeted or intracellular delivery options. However, further optimization is necessary to overcome limitations related to maintaining activity, specificity, and in vivo validation. The effects of these fusion strategies on pharmacokinetics, tissue targeting, and protein stability provide a rationale for adapting these approaches to L-ASNase-based therapeutics. Among reported constructs, tandem fusion is the most common strategy for L-ASNase-based fusion proteins and generally preserves L-ASNase enzymatic activity. In these constructs, linkers are typically flexible and relatively short (15–30 aa) or absent. Several classes of partner proteins, except immunokinases, described in Section 3, have been explored for L-ASNase fusion proteins. Although antibodies and their derivatives are among the most frequently investigated partners, despite their theoretical advantage for targeted delivery, most antibody-based L-ASNase fusions remain experimental, and some demonstrate reduced enzymatic activity compared with non-targeted constructs.

Compared with free L-ASNases (see Table 1), certain fusion proteins showed prolonged half-lives. The ABD-fused L-ASNase exhibited a 6–23-fold increase in half-life (6.83 ± 0.13 days; 163.92 ± 3.12 h). Another fusion (L-ASNase-ELP90) showed a 1.1–75-fold increase (22.00 ± 1.74 days; 528.10 ± 41.64 h) relative to PEGylated L-ASNases and free L-ASNase, respectively (see Table 1). Since there is currently little or no data on other fusion L-ASNases, it is conceivable that fusion with long polymers such as ELP and PEG or proteins like ABDs may also increase the half-life. One promising direction is the use of fusion proteins of L-ASNase with CPP (e.g., 30Kc19 [110], TAT [111]), which facilitate penetration into the cell and local and long-term (as in the case of L-ASNase-ELP) depletion of asparagine. Unlike targeted fusion of L-ASNase proteins with antibodies, fusion with CPPs preserves enzymatic activity and may facilitate the intracellular release of L-ASNase after uptake. This could potentially reduce steric constraints and affect intracellular activity.

Some fusion proteins demonstrated reduced immunogenicity compared to the free and PEGylated forms of L-ASNase. Immunogenicity has currently been studied for L-ASNase fused to ELP [100,101] and CETP [103]. Due to their relatively high molecular weight, L-ASNase fused to ELP60 and ELP90 exhibited reduced immunogenicity compared to PEGylated L-ASNases, likely due to the wrapping of the antigenic epitopes of L-ASNase. IgG and IgM titers against the PEG polymer in PEG-L-ASNase were 21-fold and 31-fold higher, respectively, than the corresponding titers against ELP60 in L-ASNase-ELP60. Similarly, they were 13-fold and 22-fold higher, respectively, than the corresponding titers against ELP90 in L-ASNase-ELP90 [100,101]. The absence of linkers (see Table 2) may also contribute to reduced exposure of potentially immunogenic sequences; however, this hypothesis requires experimental validation.

In another case, the goal was to increase the immunogenicity of L-ASNase and to create a prototype vaccine against atherosclerosis. For example, fusing L-ASNase with the B-cell epitope of CETP yielded a fusion protein whose antibodies were detectable in the blood of mice even after 18 days and which, on the other hand, had no toxic effect on the kidneys. This strategy can be used to create chimeric proteins against human autoantibodies [103].

Among the fusion proteins demonstrating promising in vivo activity, ErA II L-ASNase fused to the SA21 peptide (an ABD motif, 18 a.a.) and SUMO produced no ALL recurrence for 204 days and exhibited reduced toxicity in mice [96]. In addition, WsA L-ASNase fused to a heparin-binding sequence achieved 100% survival of mice bearing T-cell lymphoma L5178Y at day 60 [112].

With regard to solid tumors, the highest in vivo antitumor efficiency was demonstrated by L-ASNase ErA II fused with the ABD motif (48 a.a.) against gastric cancer (tumor decreased by 65%) [97] and L-ASNase EcA II with ELP90 against melanoma. L-ASNase-ELP90 exhibited greater cytotoxicity than L-ASNase or cisplatin in cancer model mice with oral cancer, melanoma, or cervical cancer. The median survival of melanoma-bearing mice in the L-ASNase-ELP90 group was 70 days, which is 2.3 times longer than in the aPD-1 group (31 days). However, unlike the two aforementioned fusion proteins, metastases appeared on day 60 in the L-ASNase-ELP90 group (compared to day 85 when combined with aPD-1) [102]. These findings suggest that prolonging systemic asparagine depletion alone may be insufficient to prevent tumor progression, particularly in heterogeneous solid tumor models. Notably, fusion proteins derived from L-ASNases and antibody fragments have been tested in mouse models. Therefore, targeted in vivo studies are required to evaluate their pharmacokinetics, efficacy, and safety.

### 4.5. Current Challenges and Future Perspectives

According to the literature, only around 20 L-ASNase-based fusion proteins have been reported to date. The main advantages of several obtained L-ASNase-based fusion proteins obtained to date over other strategies (e.g., PEGylation, erythrocyte encapsulation, or nanoparticle delivery [114]) are a prolonged half-life, reduced immunogenicity, and potentially improved antitumor efficacy. For example, L-ASNase-ELP has shown lower immunogenicity and longer circulation than PEGylated L-ASNase, while engineered fusions such as ErA II–SA21 and WsA fused to a heparin-binding sequence demonstrated enhanced therapeutic efficacy in ALL and T-lymphoma L5178Y mouse models, respectively (survival was 100% at day 204 and day 60, respectively), and L-ASNase-ELP in melanoma models (survival was 100% after 70 days [102]). Previously, several free L-ASNases have shown in vivo efficacy in ALL and solid tumors:

− Pegaspargase (PEGylated EcA II)—active against ALL; median survival—63–67 days; % of survival not reported [115];
− Crisantaspase (PEGylated ErA II)—active against ALL; median survival—51–56 days; % of survival not reported [116];
− Crisantaspase—active against hepatocellular carcinoma; study duration 3 weeks; median survival not reported [117].
− EcA II encapsulated in erythrocytes—active against pancreatic ductal adenocarcinoma; study details were not specified [118].
− Humanized L-ASNase EBD-200—tested in melanoma (30-day study) and liver cancer (45-day study); median survival not reported [119].

EcA II encapsulated in nanoparticles with rotenone—tested against triple-negative breast cancer (median survival 86 days; 4/6 mice survived) and colorectal cancer (median survival 66 days; 5/6 mice survived) [120].

While the anticancer activity of these enzymes was evaluated in different tumor models and study designs, direct comparisons and definitive conclusions about their relative efficacy are rather complicated.

The potential limitations of these fusion proteins include their limited efficacy against certain hematological and solid tumors due to resistance mechanisms against L-ASNases [121], either alone or in combination with other drugs, as well as the formation of metastases [102]. Another potential limitation is the increased molecular size of some fusion proteins, which may affect tissue penetration, including penetration into solid tumors and across the blood–brain barrier. Additionally, there is a risk of increased immunogenicity due to the multiple components of the fusion protein, which can trigger immune reactions and side effects (Table 3).

The main challenges and promising directions for developing fusion L-ASNases are as follows:

(1) Increasing the solubility and activity of fusion constructs by optimizing expression systems and tag/linker design. This includes selection and optimization of plasmids and promoters, the incorporation of solubility-enhancing tags (e.g., SUMO, MBP, Trx, FLAG, etc.), the design and testing of different linkers (flexible, rigid, cleavable) and combinations of these within single constructs [122].
(2) Purification and production. Compared to free-form L-ASNase, the production of fusion proteins may require several purification steps, which can lead to increased costs [3].
(3) Implementing in silico prescreening structure and stability. Using bioinformatic and computational tools (AlphaFold, SWISS-MODEL, Rosetta, GROMACS, FoldX, molecular docking and others) to assess stability, folding, interaction interfaces and potential aggregation. For example, the structure of two drug candidates, L-ASNase-ELP60 and L-ASNase-ELP90, was calculated in AlphaFold2.
(4) Extending circulation time. Potential strategies include fusion to half-life–extending domains or peptides (e.g., ELPs, ABDs, scFvs, and nanobodies) or post-translational conjugation to polymers. It is known that even drugs with an extended half-life can be immunogenic. Therefore, extensive pharmacokinetic and pharmacodynamic studies of such proteins are necessary to prevent any potential drawbacks and side effects [101].
(5) Immunogenicity. It is well established that the most widely used L-ASNAse, EcA II, is frequently associated with adverse effects, including hypersensitivity (including anaphylaxis), hepatotoxicity, and thrombosis [10]. Even in the absence of overt clinical reactions, anti-EcA II antibodies may develop, reducing therapeutic efficacy. Use of PEGylated EcA II is also limited in patients who mount immune responses, because PEGylation does not eliminate antigenic epitopes and the effective half-life can be reduced by immune clearance [123]. EcA II is currently the most widely used fusion L-ASNase (see Table 2) and remains a frequent cause of hypersensitivity. One approach to reducing the immunogenicity of L-ASNases is the use of fusion proteins with low immunogenicity that lack linkers, as these can also potentially trigger immune reactions. ELPs used as fusion partners have been reported to substantially decrease immunogenicity [100]. An alternative approach is to use L-ASNases from other microbial sources that differ in epitope arrangement and amino-acid composition. There is also evidence that specific engineered constructs show improved safety and efficacy. The fusion of an ABD motif to an ErA II mutant (A31I/E63Q/S254Q) [97] and the fusion of a WsA mutant to a heparin-binding sequence [112] have both been reported to reduce toxicity. Early, reduced immunogenicity has been observed for enzymes such as ErA II, WsA, *Erwinia carotovora* (EwA), and *Rhodospirillum rubrum* (RrA) [123]. Immunogenicity can also be reduced by targeted mutagenesis. For example, the N24A/Y250L mutant of L-ASNase EcA II had reduced glutaminase activity [124]. Another way to reduce immunogenicity is to identify and modify antigenic residues in the enzyme. For example, the immunogenicity of L-ASNase EcA II decreased due to the R195A/K1961/H197A mutations [22]. Using bioinformatic approaches to identify immunogenic epitopes in L-ASNases shows promise. These approaches can be complemented by structural predictions with immunogenicity and allergenicity screening (e.g., ProtParam, ToxinPred, VaxiJen, NetMHCII 4.0, AllerTOP, etc.) or identification of epitopes (Discotope, ElliPro and EPSVR or the Immune Epitope Database server) [85,123,125]
(6) Enhancing efficacy and tumor targeting. Currently, the clinical use of L-ASNase is limited to ALL and non-cystic T-cell lymphoma, as these diseases exhibit reduced expression of asparagine synthetase (ASNS), which catalyzes the conversion of L-aspartate and L-glutamine to L-asparagine and L-glutamate. Therefore, most cases of ALL and NCTCL cases are asparagine auxotrophic, necessitating treatment with L-ASNase [126].
  Designing targeted fusion proteins capable of receptor-mediated delivery and/or intracellular penetration to overcome resistance to L-ASNase-induced apoptosis (e.g., fusion with scFvs against tumor antigens; HBDs; TRAIL domains; or CPPs). Comparing their efficacy with that of free form of the L-ASNase. One promising approach is to create CAR-T cells with chimeric proteins based on L-ASNase. Recently, CAR-T cells were modified using vectors containing the L-ASNase gene and scFvs to reprogram asparagine metabolism and overcome resistance to immunotherapy in refractory ALL [127]. The use of fusion L-ASNases to treat some solid tumors with low ASNS expression is also promising. Despite the development of resistance mechanisms, including autophagy, hyperactivation of the GCN2-eIF2-ATF4 [128], MAPK [129], or KRAS/PI3K/AKT/mTORC1 [130] signaling pathways, macropinocytosis, autophagy, and mutations in the BRAF [131] or KRAS [132] genes, several approaches to overcoming resistance have been developed. These approaches could be used in combination with fusion L-ASNases or to design fusion proteins targeting specific components of these signaling pathways and molecules. Identifying mechanisms of resistance to L-ASNase has stimulated research into promising new drug combinations, such as the use of GCN2 inhibitors (e.g., ibrutinib), could be used to suppress the GCN2-ATF4 axis, which is responsible for the re-induction of ASNS expression in asparagine deficiency. Such combination therapy may be effective in treating ALL, AML, pancreatic cancer, and melanoma. Additionally, several strategies have been developed to enhance tumor sensitivity to L-ASNase action in cases of MAPK signaling pathway hyperactivation. Depleting L-asparagine in combination with suppressing the MAPK signaling pathway using various inhibitors has been shown to effectively suppress melanoma growth and metastasis in vivo [128]. A phase I clinical trial (NCT05034627) is currently underway to investigate the possibility of using the MEK inhibitor cobimetinib in combination with calaspargase in patients with pancreatic cancer [21]. Another approach being developed to overcome resistance to L-ASNase is the effect on immune checkpoints [102]. The checkpoint inhibitor has already been used in combination with the L-ASNase-ELP90 fusion. This combination increased the median survival of mice from 70 days to 85 days [102].
(7) In vivo validation. Further long-term studies in relevant leukemia and solid tumor models are required to establish the therapeutic efficacy, safety, pharmacokinetic properties, and immunogenicity of L-ASNase fusion constructs (see Table 2). Additionally, systematic comparative studies are required to determine which targeting and effector domains provide the optimal balance between tumor specificity, enzymatic activity, and safety. This represents an important area for the future optimization of L-ASNase-based fusion therapeutics.

## 5. Conclusions

Protein fusion technology has emerged as a promising strategy for overcoming the limitations of L-ASNase, such as its short half-life, immunogenicity, and lack of tumor specificity. Compared with traditional approaches such as PEGylation, fusion-based designs offer several advantages, including a longer circulation time, targeted delivery, and, in some cases, reduced side effects.

Among the reviewed strategies, fusion with ELPs is particularly effective at extending half-life to up to 528 h—compared to approximately three hours for native L-ASNase—while preserving most of the enzyme’s activity. In animal models, such constructs improve therapeutic outcomes, with a twofold increase in survival compared to native L-ASNase. However, fusion to antibody-derived fragments (e.g., scFvs or nanobodies) enables targeted delivery and improved specificity but is often associated with reduced catalytic or cytotoxic activity, which may limit overall therapeutic efficacy. Other targeting or cytotoxic approaches, including CPPs, HBDs, and TRAIL-based fusions, further expand the potential applications of L-ASNase, particularly beyond hematological malignancies. Notably, some of these approaches use low-molecular-weight targeting domains, which enable prolonged half-life and efficacy without the need for solubility tags or long linkers.

Despite these advances, several challenges remain. These include potential loss of enzymatic activity, limited long-term efficacy in vivo (e.g., metastatic progression), and the complexity of designing and producing fusion proteins. Additionally, although current computational tools have improved significantly (e.g., AlphaFold), they remain insufficient for predicting the behavior of complex fusion constructs, especially those containing flexible linkers. Thus, experimental validation remains essential.

Future research should focus on the systematic optimization of fusion partners and linker design, and the development of multifunctional constructs that combine targeting and half-life extension capabilities. Overall, the engineering of fusion proteins represents a promising approach to developing the next-generation of L-ASNase therapeutics with improved pharmacological properties and broader applicability to both hematological and solid tumors.

## Figures and Tables

**Table 1:** Characteristics of clinically approved L-ASNases.

L-ASNase	Specific Activity (IU/mg Protein)	Elimination Half-Life in Serum
EcA II	~300–600 [9,10]	25.8 ± 9.9 h (Spectrila^®^) [11]; 30.7 ± 8.4 h (Elspar^®^) [12]
ErA II	~330–350 [9,10]	7.5 h (intravenous) or 15.6 (intramuscular) [13]
PEG-EcA II	~233 [14] to 750 (Oncaspar^®^) [15]	5.7 ± 3.2 days (136.8 ± 76.8 h) (Oncaspar^®^) [16]; 16.1 days (386.4 h, Asparlas^®^) [17]
PEG-ErA II	~640 [18]	20 days (480 h) [18]

**Table 2:** Comparative summary of the properties of L-ASNase fusion proteins.

L-ASNAse	Partner Peptide/Protein	Linker Between L-ASNase and Partner	Vector	*E. coli* Expression Strain	Induction Temperature and IPTG Concentration	Solubility	Specific Activity	Half-Life	In Vitro Cytotoxicity (IC_50_)	In Vivo Efficacy	Reference
EcA II	Anti-EcA scFv of mAb#12-4	2 × (G4S)3	pTrcHisA	Not provided	37 °C	inclusion bodies	13.3 IU/mg	-	-	-	[91]
EcA II	Anti-EcA scFv46	(G4S)6	pET 21a	BL21 (DE3)	30 °C; 1 mM	soluble	102 U/mg	9 h	-	-	[93]
EcA II	anti-CD19 scFv	6 × GGGGS	pET45b(+)	Origami (DE3) with the pTfl6 plasmid	10 °C; 1 mM	soluble	90.9 ± 12.9 U/mg	-	0.002 and 0.004 U/mL for RS4 cells and 0.48 and 0.50 U/mL for Raji	-	[107]
Truncated form of EcA II (12 amino acids from active site)	anti-CD19 scFv	3 × GGGGS	pET45b(+)	BL21(DE3)	25 °C; 1 mM	inclusion bodies	4.30 ± 0.26 U/mg	-	0.13 ± 0.02 U/mL for MOLT-4 cells	-	[95]
ErA II mutant (A31I/E63Q/S254Q)	SUMO, ABD (SA21 peptide, 18 a.a.)	lacked	not provided	not provided	18 °C; 0.3 mM of IPTG	soluble	417.7 ± 13.6	38.3 h	-	There was no recurrence of ALL within 204 days; reduced toxicity in mice	[96]
ErA II	ABD (46 a.a.)	(GGGGS)5	pET30a	BL21(DE3)	16 °C; 0.2 mM of IPTG	soluble	399.7 IU/mg	6.83 ± 0.13 days	0.11 μg/mL for MKN-45 cells	Gastric tumor was reduced by ~65% in volume and ~61% in weight after 6 weeks; absence of acute toxicity	[97]
EcA II	ELP60 or ELP90	lacked	pET25b(+)	Rosetta-gami (DE3) pLys	not provided	soluble		191 h; 353.2 h		The median survival time of leukemia mice treated with EcA II-ELP90 (~80 days) was 1.25 and 2.12 times longer than that of mice treated with EcA II-ELP60 and EcA II.	[100]
EcA II	ELP90	lacked	pET25b(+)	Rosetta-gami (DE3) pLys	not provided	soluble	(0.8 IU/nM)	528.10 ± 41.64 h	IC_50_ for fused protein was comparable with native EcA II for A375, B16F10, HeLa, HN6, 4T1, OVCAR3, HepG2, HL-7702, A431, CT26	The median survival time of mice with melanoma treated with EcA II-ELP90 was 2.3 times longer (70 days) than those in the aPD-1 group (31 days)	[102]
Guinea pig L-ASNase 1 and human L-ASNase chimera	Arginase I	A(EAAAK)2A	pET22b(+)	ArcticExpress (DE3)	20 °C; 0.01 M	inclusion bodies	2.69 IU/mL (0.0517 IU/mg)	-	-	-	[105]
EcA II	Ser-Gly-Thr	TTP, CETP	pET28	BL21(DE3)	37 °C; 5 mM lactose	soluble	200 U/mg	-	-	Strong CETP-immune response in mice, absence of pathological changes in the kidneys	[103]
EcA II	30Kc19	PLGLAG	pET23	BL21(DE3)	37 °C; 1 mM	soluble	11.43 IU/mL	-	Selective cytotoxicity for L5178Y cells and ability to penetrate into L5178Y cells. IC_50_ was 3 mU/mL.	-	[110]
EcA II	TAT (CGGGYGRKKRRQRRR)	Chemical disulfide bond	-	-	-	soluble	124 ± 20	-	IC_50_ was 0.0102 against MOLT-4 cell	Increases the average survival time of mice with T-lymphoma L5178Y from 13.9 (native L-ASNase) to 15.6 days.	[111]
WsA mutant (V23Q/K24T)	Heparin-binding sequence (KRKKKGKGLGKKR)	Pch PRP8 intein with Cys1Ala and Cys8Tyr mutations	pET28b(+)	BL21(DE3)	37 °C; 0.14 mM	soluble	187 ± 12	-	effectively bound to the surface of K562 cells by heparin-binding site	Single dose of 1000 or 2000 IU/kg in a 5-fold regimen, 100% survival of mice with T-lymphoma L5178Y was observed	[112]
ErA II mutant (A31I/E63Q/S254Q)	His-SUMO- FOLDON-TRAIL	GGGS(GGGGS)3	pET14b	BL21(DE3) C41	37 °C; 0.3 mM	soluble	not provided	-	IC_50_ was 0.064 IU/mL for AML MV4 cells	High cytotoxic activity in mice with MV4 (data not provided)	[83]

**Table 3:** Main advantages and current limitations of L-ASNase fusion proteins based on different fusion partners.

Fusion Partner	Main Advantage	Current Limitation
ABD	Extended half-life while maintaining enzymatic activity	No clinical validation
ELP	Extended half-life and reduced immunogenicity demonstrated in preclinical models	Non-targeting
scFv, nanobody	Targeted delivery; compact size	Often reduced activity; limited in vivo validation
CPP (30Kc19, TAT)	Enhanced intracellular delivery	Limited tumor specificity and potential systemic toxicity
HBD	Tumor-associated targeting and improved in vivo efficacy	Limited clinical validation
TRAIL	Enhanced cytotoxic activity through apoptosis induction	Only in vitro experimental data

## Data Availability

No new data were created or analyzed in this study.
